# A novel microfluidic device capable of maintaining functional thyroid carcinoma specimens ex vivo provides a new drug screening platform

**DOI:** 10.1186/s12885-019-5465-z

**Published:** 2019-03-22

**Authors:** Andrew Riley, Victoria Green, Ramsah Cheah, Gordon McKenzie, Laszlo Karsai, James England, John Greenman

**Affiliations:** 1 Faculty of Health Sciences, University of Hull, Kingston upon Hull, HU6 7RX UK; 2 Hull and East Yorkshire Hospitals NHS Trust, Kingston upon Hull, HU16 5JQ UK; 3 Hull York Medical School, University of Hull, Kingston upon Hull, HU6 7RX UK

**Keywords:** Thyroid gland, Microfluidics, Viability, De-differentiation, Radioiodine therapy

## Abstract

**Background:**

Though the management of malignancies has improved vastly in recent years, many treatment options lack the desired efficacy and fail to adequately augment patient morbidity and mortality. It is increasingly clear that patient response to therapy is unique to each individual, necessitating personalised, or ‘precision’ medical care. This demand extends to thyroid cancer; ~ 10% patients fail to respond to radioiodine treatment due to loss of phenotypic differentiation, exposing the patient to unnecessary ionising radiation, as well as delaying treatment with alternative therapies.

**Methods:**

Human thyroid tissue (*n* = 23, malignant and benign) was live-sliced (5 mm diameter × 350-500 μm thickness) then analysed or incorporated into a microfluidic culture device for 96 h (37 °C). Successful maintenance of tissue was verified by histological (H&E), flow cytometric propidium iodide or trypan blue uptake, immunohistochemical (Ki67 detection/ BrdU incorporation) and functional analysis (thyroxine [T4] output) in addition to analysis of culture effluent for the cell death markers lactate dehydrogenase (LDH) and dead-cell protease (DCP). Apoptosis was investigated by Terminal deoxynucleotidyl transferase dUTP nick end labelling (TUNEL). Differentiation was assessed by evaluation of thyroid transcription factor (TTF1) and sodium iodide symporter (NIS) expression (western blotting).

**Results:**

Maintenance of gross tissue architecture was observed. Analysis of dissociated primary thyroid cells using flow cytometry both prior to and post culture demonstrated no significant change in the proportion of viable cells. LDH and DCP release from on-chip thyroid tissue indicated that after an initial raised level of release, signifying cellular damage, detectable levels dropped markedly. A significant increase in apoptosis (*p* < 0.01) was observed after tissue was perfused with etoposide and JNK inhibitor, but not in control tissue incubated for the same time period. No significant difference in Ki-67 positivity or TTF1/NIS expression was detected between fresh and post-culture thyroid tissue samples, moreover BrdU positive nuclei indicated on-chip cellular proliferation. Cultured thyroid explants were functionally viable as determined by production of T4 throughout the culture period.

**Conclusions:**

The described microfluidic platform can maintain the viability of thyroid tissue slices ex vivo for a minimum of four days, providing a platform for the assessment of thyroid tissue radioiodine sensitivity/adjuvant therapies in real time.

## Background

Thyroid carcinoma (TC) is the most common malignant disease of the human endocrine system, accounting for ~ 2.1% of all cancer cases worldwide, and disproportionately affecting women [1]. The incidence of TC has increased by 139% since the early 1990’s in the United Kingdom, with a particular increase in the diagnosis of papillary TC (PTC; Cancer Research UK [2018]). PTC, along with follicular-TC (FTC), form the differentiated TC group (DTC), which represent ~ 90% of all thyroid malignancies [2]. Patients with DTC have a 10-year survival rate of 90%, underpinned by an efficacious treatment regimen, which includes thyroidectomy followed by remnant and metastatic ablation using radioactive iodine (^131^I).

Treatment, using ^131^I, capitalises on the ability of thyrocytes to absorb iodide (I^−^) for thyroid hormone biosynthesis. I^−^ is transported across the basolateral membrane of thyrocytes by the sodium iodide symporter (NIS) using the sodium gradient generated by Na^+^/K^+^-ATPase [3]. Due to the essential involvement of the NIS in thyroid hormone biosynthesis and thus human metabolism in general, expression of the NIS is tightly regulated. In thyroid cancer, thyroid peroxidase (TPO) activity, and thus organification of free iodide is reduced. Oxidised iodine possesses a significantly longer retention time than free iodide, leading to a considerably reduced effective half-life in the malignant compared with the benign thyroid (0.5–3 days vs. 3–7 days, respectively; [4]). Despite this, treatment of DTC using ^131^I remains an effective means of eradicating remnant and metastatic malignant foci when a high dose can be delivered to the target tissue [5]. However, in approximately 15% of DTC cases, basolateral expression of the NIS is lost, due to phenotypic de-differentiation of thyrocytes, rendering ^131^I therapy ineffective [6]. Within this cohort of patients, the use of ^131^I therapy is negated, as thyrocytes cannot concentrate ^131^I, leading to a marked reduction in patient survival (only 10% alive at 10-years, mean survival 3–5 years; [7]). Treatment of advanced, ^131^I refractory TC using traditional systemic therapies including doxorubicin has limited efficacy with a high toxicity rate [8]. Alternative therapy using relatively new-to-market tyrosine-kinase inhibitors (TKI), particularly sorafenib and lenvatinib, has garnered significant interest and has shown objective response rates with significantly improved progression-free survival [9, 10]. Currently it is not possible to predict accurately a lack of response to ^131^I prior to administration, thus patients have a delay before receiving effective adjuvant therapy [11]. Although it is well accepted that iodide uptake is typically reduced in malignant thyroid tissue relative to surrounding tissue [12], the molecular underpinnings have not yet been fully elucidated. A previous study by Min et al. [6] investigated the relationship between NIS expression and ^131^I uptake in recurrent TC using immunohistochemical (IHC) analysis. They found that although lesional NIS expression predicted ^131^I uptake with 100% specificity, the sensitivity of this method was just 50% (14 of 28 patients with positive ^131^I uptake had negative NIS staining. Similarly, Arturi et al. [13] found, in a smaller study using RT-PCR, that only 4 out of 8 (50%) primary DTC with distant metastases and negative ^131^I uptake lacked NIS gene expression in the primary lesion; thus indicating that assessment of NIS expression is certainly not an infallible means of evaluating ^131^I uptake. Furthermore, Saito et al. [14] unexpectedly detected increased NIS mRNA and protein levels in PTC tissue compared with normal thyroid tissue. This variability suggests that reduced iodide uptake is perhaps due to failure to target or retain NIS protein at the basolateral membrane [15], rather than abrogated NIS protein production.

The microfluidic system described herein uses precision cut tissue slices (PCTS) which maintain the multicellular 3D architecture of the thyroid tissue essential for intercellular communication, which is not well recapitulated in standard in vitro systems. Two previous studies have employed static culture of thyroid tissue explants: Russo et al. [16] investigated apoptosis subsequent to ^131^I treatment in 1mm^3^ tissue fragments, whereas Nagy et al. [17] examined the effect of various hormones and cytokines on the proliferation of 2mm^3^ thyroid fragments. The results from these studies demonstrated that thyroid tissue cultured ex vivo can respond to external stimuli, however clinical utility was not assessed.

The bespoke device described in this manuscript mimics the in vivo vasculature and lymphatics by allowing continual perfusion of explanted tissue, along with removal of waste products and allows precise control of drug delivery [18]. Future treatment of ex vivo thyroid tissue maintained in this device with ^131^I, holds potential for a more realistic estimation of ^131^I treatment success by assaying not only uptake, but resultant cell death. The aim of this study was to characterise a reproducible, easy to use, microfluidic system to maintain precision sliced ex vivo thyroid tissue for up to 96 h.

## Methods

### Fabrication of a microfluidic culture device

**Fig. 1:**
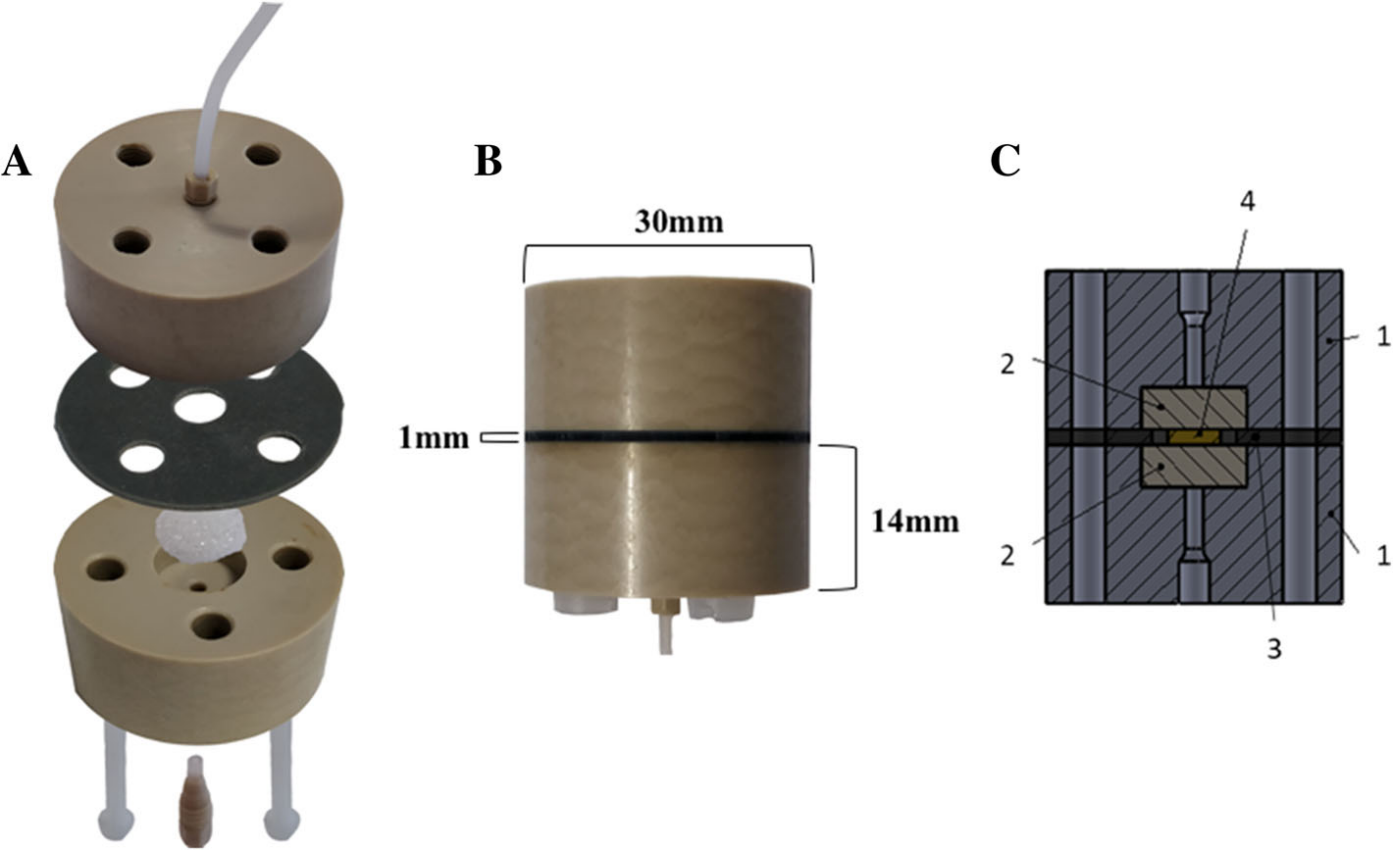
PCTS Culture device. **a**: Photograph illustrating device assembly. Two polyether ether ketone (PEEK) plastic plates, each with a medial recess to house a sintered pyrex disk, affix to sandwich a silicone gasket with a central cavity in which the precision cut tissue slice (PCTS) is contained. A 70 μM porous nylon membrane separates the tissue from the sintered disk (not shown). **b**: Photograph of an assembled PCTS culture device with external dimensions. **c**: Schematic of PCTS culture device. Component parts numerically labelled: 1, PEEK plastic plates; 2, glass sintered pyrex disk; 3, central silicone gasket; 4, tumour tissue sample

Two polyether ether ketone plastic plates (PEEK; Direct Plastics, Sheffield, UK), both 30 mm × 14 mm in size (Fig. 1-a), were milled centrally to produce threaded axial holes to which inlet and outlet ethylene tetrafluoroethylene tubing (ETFE; 0.8 mm internal diameter; Kinesis, IDEX Health & Science, Cambridge, UK) was attached via coned adapters (LabSmith, Mengel Engineering, Denmark). In addition, four ¼ inch (6.35 mm) holes were made in both PEEK plates to allow the insertion of nylon screws in order to secure the unit after sample addition. A central cylindrical recess (10 × 4 mm) was drilled in each PEEK plate to house a porous sintered pyrex disc (Fig. 1-a; The Lab Warehouse, Grays, UK). Finally, a silicone gasket (30 mm diameter, 1 mm sheet silicone) with a 6 mm central hole to create a tissue well, was placed between the two PEEK plates.

### Sample collection and preparation

Human thyroid tissue samples (*n* = 23 malignant; *n* = 14 benign) were collected at the point of surgical resection during thyroidectomy following written informed consent under ethical approval from North East-Newcastle and North Tyneside Research Ethics Committee (15/NE/0412) and Hull and East Yorkshire NHS Trust R&D (R1925). Benign thyroid tissue was collected alongside malignant tissue when deemed practicable by consultant surgeon JE (n = 14). Details of obtained tissue specimens are summarised in Table 1. Tissue samples were transported to the laboratory in ice-cold complete medium, then processed, allowing culture initiation within 60 min of surgical excision. Tissue was immobilised on a piece of cork and sliced at a thickness of either 350 or 500 μm in ice-cold PBS using a vibratome (Leica VT1200S, Milton Keynes, UK) with a blade speed of 0.1 mm s^− 1^ and amplitude of 2.5 mm. A skin biopsy punch (Stiefel, Middlesex, UK) was used to generate PCTS 5 mm in diameter. Each PCTS was weighed before insertion into the device. Where samples sizes differ across experimental time-points this was due to difficulties in effluent collection, sample numbers collected are provided in figures.

**Table 1:** Disease and patient characteristics of thyroid specimens used in this study

No	Tumour stage	Patient age range (years)	Tissue received
1	T2NXMX	40–50	M
2	T4aN1bMX	40–50	M
3	T3aNXMX	40–50	M
4	BG	50–60	B
5	T2NXMX	50–60	M,B
6	T3N1bMX	20–30	M
7	T4N0M0	80–90	M
8	T1aNXMX	60–70	M
9	BG	60–70	B
10	T2N1BMX	30–40	M,B
11	T3N1aMX	50–60	M
12	BG	30–40	B
13	BG	50–60	B
14	T4N0M1	50–60	M
15	T1aN1bMX	40–50	M
16	T1bNXMX	40–50	M,B
17	T3aNXMX	10–20	M
18	T3bN1aMX	40–50	M,B
19	T1NXMX	60–70	M,B
20	T1aNXMX	10–20	M
21	T3AN1bMX	30–40	M,B
22	T3aN1bM0	20–30	M
23	T1aNXMX	40–50	M,B
24	T3N1bM0	30–40	M,B
25	T2NXMX	70–80	M,B
26	T3aNXMX	60–70	M
27	T4aN1bMX	70–80	M,B

*BG* Benign goitre, *M* Malignant tissue, *B* Benign tissue

### PCTS culture set up

Dulbecco’s modified eagles medium (DMEM; GE Healthcare, Yeovil, Somerset, UK) containing 10% (*v*/v) heat inactivated foetal bovine serum (FBS; Biosera, East Sussex, UK), penicillin/streptomycin (0.1 U/ml and 0.1 mg/ml respectively, GE healthcare), 0.4 mM glutamine (GE healthcare) and 2.5 μg/ml Amphotericin B (Life Technologies, Paisley, UK) was supplemented with thyrotropin [TSH; 2 mIU/L (normal serum 0.3–5 mIU/L)] and sodium iodide (0.1 μg/ml). ‘Complete medium’ was then loaded into a 20 ml syringe and connected to the 2-part adapter and ETFE tubing via a 0.22 μm filter (Fig. 1; Millipore, Watford, UK). The PCTS was loaded onto a 70 μm nylon porous membrane placed on top of the sintered disc in the inlet PEEK plate, and the outlet PEEK plate was then secured in place using nylon screws. The syringe was connected to a Harvard PhD 2000 syringe pump (Harvard, Cambridge, UK), which provided a pressure driven perfusion rate of 2 μl min^− 1^. The culture device was maintained at 37 °C inside an incubator for 96 H*. media* coming off the chip was collected in 1.5 ml polypropylene tubes after 2 h culture, then in 15 ml polypropylene tubes once per day thereafter. Where required, for proliferation analyses, the thymidine analogue Bromodeoxyuridine (BrdU; Sigma-Aldrich, Gillingham, UK) was added, at 10 μM final concentration, to the complete medium for the final 24 h of culture [19].

### Tissue embedding and morphological analysis

Fresh and post-culture tissue samples were either frozen in Tissue-Tek OCT (optimum cutting temperature; Sakura, Berkshire, UK) medium, using liquid nitrogen-cooled 2-methylbutane (Sigma-Aldrich), or fixed in 4% paraformaldehyde (PFA) for 24 h. PFA fixed tissues were then dehydrated through an ethanol gradient (70, 90, 95, 100%), incubated with two changes of molten paraffin wax, then embedded and allowed to cool. Frozen tissue sections were cut at a thickness of 8 μm using a Leica CM1100 cryostat and fixed for 20 min in − 20 °C cooled methanol before standard staining using Haematoxylin and Eosin (H&E; [20]). PFA fixed, paraffin embedded (PFPE) tissues were sectioned using a Leica RM2135 microtome (5 μm).

### Measurement of lactate dehydrogenase release

Lactate dehydrogenase (LDH) is a cytosolic enzyme released from the cell after plasma membrane damage [21]. As membrane damage is synonymous with cell death, its release allows monitoring of the loss of cell viability (Cytotoxicity Detection Kit Plus, Roche, UK). Culture effluent (*n* = 14 malignant, *n* = 10 benign patient samples) was collected daily and stored at 4 °C until the end of the culture period (96 h). In some cases (*n* = 7) tissue was removed from the culture system at the end of the 96 h incubation and exposed to a 10% (*v*/v) lysis buffer overnight to induce cell death and subsequent LDH release. The assay was conducted following the manufacturer’s protocol and results expressed as an average of duplicate readings normalised per mg of thyroid tissue.

### Measurement of protease release

The CytoTox-Glo™ Cytotoxicity Assay (Promega, Southampton, UK) allows measurement of cell death using a luminogenic peptide substrate (alanyl-alanylphenylalanyl-aminoluciferin; AAF-Glo™ Substrate) to measure the activity of proteases released from non-viable cells that have lost membrane integrity. Culture effluent samples (including positive controls exposed to 10% (*v*/v) lysis buffer; 100 μl per well) were added in duplicate to a 96-well white-walled microplate. Malignant tissue samples were prioritised for use in DCP analysis. Assay Reagent (50 μl) was then added to all wells and mixed briefly by orbital shaking. The reaction was incubated at room temperature for 15 min, before luminescence was measured using a Victor multilabel plate reader according to the supplier’s protocol (*n* = 7; PerkinElmer, Coventry, UK).

### Viability analysis of dissociated cells

Malignant thyroid tissue was dissociated into a single cell suspension (*n* = 3) prior to and after maintenance on the device by initial mincing using scalpels followed by a 2 h incubation (37 °C; 5% CO_2_) with 0.024% (*w*/*v*) Collagenase IV (Sigma-Aldrich: Dorset, UK) and 0.02% (w/v) DNase I (Roche, Herefordshire, UK) in complete medium under constant rotation. The resultant suspension was passed through a 70 μm filter (BD Biosciences, Oxford, UK) prior to centrifugation at 400 x *g* for 5 min, to pellet the cells. The cell pellet was resuspended in 1 ml of complete medium before viability was quantified using trypan blue exclusion. The cells were rinsed in PBS (Oxoid, Thermo Scientific, Hampshire, UK)/ BSA (2.5 g/L; Thermo Scientific, Loughborough, UK)/Azide (0.0624% [*v*/v]; Sigma-Aldrich) and dead cells were stained by the addition of propidium iodide (500 μg ml^− 1^). Cells were acquired immediately using a FACS Calibur flow cytometer (BD Biosciences) alongside unstained cells as a reference and results were analysed using Cell Quest Pro software, version 6.0.

### Terminal deoxynucleotidyl transferase-mediated dUTP nick-end labelling (TUNEL) assay

Frozen tissue sections were defrosted for 5 min prior to fixing in 4% PFA for 20 min at room temperature. PFPE tissue sections were dewaxed in xylene (20 min) then rehydrated in 100, 90 and 70% ethanol (2 min per wash). Sections were treated for 20 min at 37 °C (PFPE tissue) or 2 min on ice (frozen tissue) with 0.1% proteinase K/ 1% TrisHCl in dH_2_0. Sections were washed twice with PBS for 5 min and apoptotic cells were labelled using an In Situ Cell Death Detection Kit (Roche, UK) according to the manufacturer’s guidelines. Negative control sections were incubated with 50 μl label solution only, whereas test sections were incubated with both the label and enzyme solution. Sections were counterstained using a DAPI-hardset mounting medium (Vector Laboratories, UK) before fluorescence was evaluated using an ImageXpress Micro 4 Imaging System (Molecular Devices, California, USA). Produced tiled images were stitched to create a single composite image before apoptotic nuclei were quantified using ImageJ (Fiji plugin [22]).

### Treatment of thyroid tissue with etoposide and SP600125

Thyroid explants were exposed to etoposide and SP600125 for the complete (96 h) culture period in order to examine the ability of the system to induce a response which was then quantifiable using TUNEL staining. Etoposide, a topoisomerase II inhibitor, and SP600125, a JNK inhibitor, were added to complete culture medium at a concentration of 20 μM, then perfused through the culture system under the same conditions as control samples.

### Immunohistochemical analyses

Frozen tissue sections (8 μm) were fixed in pre-cooled (− 20 °C) acetone for 20 min. Frozen tissue sections to be stained with anti-BrdU primary antibody were transferred into 2 M HCl for 30 min to denature the DNA and allow access of the antibody to the incorporated BrdU before being neutralised in borate buffer (0.2 M boric acid, 0.05 M sodium tetraborate [pH 8.4]; 2 × 5 min washes). PFPE tissue sections were dewaxed and rehydrated as previously described. Endogenous peroxidases were blocked in 3% (*v*/v) H_2_O_2_ (Fisher Scientific, Loughborough, UK) in acetone for 15 min. Antigen retrieval was carried out on paraffin embedded tissue sections by microwave boiling within Antigen Unmasking Solution (citrate based; pH 6.0, Vector Laboratories, Peterborough, UK) for 20 min. Slides were loaded into Sequenza™ racks and sections were incubated with normal horse blocking serum (Vectastain Elite ABC kit, Vector Laboratories) for 20 min. The addition of avidin D solution and, after a TBS wash, biotin solution (Vector Laboratories) for 15 min blocked non-specific binding of the avidin/biotin system.

Sections were incubated with primary antibody or matched isotype control antibody overnight at 4 °C [Ki67 clone MIB1, 1:100 (Dako, Agilent, Stockport, UK)]; [BrdU clone 3D4, 1:50 (BD Biosciences)]; [TTF1 clone EP1584Y, 1:250 (AbCam)]. Following a TBS wash sections were incubated with secondary antibody (Vectastain Elite ABC kit) for 30 min followed by ABC detection reagent for 30 min. Colour was developed using 3,3′-diaminobenzidine (DAB; Sigma-Aldrich) and sections counterstained with Harris Haematoxylin. Sections were dehydrated and cleared through graded alcohols and histoclear™ (Scientific laboratory supplies, Nottingham, UK) then mounted using histomount (Scientific laboratory supplies). The percentage of DAB positive cells was assessed across five separate fields using the online applet Immunoratio (http://153.1.200.58:8080/immunoratio/; [23]).

### Assessment of NIS protein expression by Western blotting

Both fresh and post-culture thyroid tissue samples were minced using scalpel blades in ice cold RIPA buffer (Sigma-Aldrich) containing 1% (*v*/v) protease inhibitors (Roche) and sonicated for 1 min before being agitated using a MACSmix™ tube rotator (2 h, 4 °C; Miltenyi Biotec, Surrey, UK). Samples were then centrifuged at 16,000 x *g* for 20 min at 4 °C, before the supernatant was aliquoted and frozen (− 80 °C). Proteins were quantified using a Pierce™ BCA Protein Assay Kit (Thermo Scientific) then 10 μg was resolved on a 4% (v/v) stacking, 12% (v/v) separating sodium dodecyl sulfate–polyacrylamide gel in a Mini-PROTEAN® system, before being transferred onto nitrocellulose membranes using a Trans-Blot Turbo™ (both Bio-Rad, Watford, UK) and blocked in 4% bovine serum albumin (BSA) in TBS-Tween overnight. NIS was detected using a rabbit anti-human polyclonal antibody (ab199410, Abcam, Cambridge, UK) diluted 1:500. The house keeping gene β-actin was detected using a mouse anti-human antibody (sc-516,102, Santa-Cruz Biotechnology, Heidelberg, Germany) diluted 1:10,000. Blots were developed using SuperSignal™ West Pico PLUS Chemiluminescent Substrate (Thermo Scientific) and Radiomat LS film (Agfa Healthcare, Middlesex, UK). Densitometry of bands was analysed using a Gel Doc system (Bio-Rad).

### Measurement of thyroxine release by ELISA

Thyroxine (T4) is the principal hormone released by the thyroid gland, thus, its release from on-chip thyroid tissue was investigated using an enzyme-linked immunosorbent assay (ELISA, Abcam), according to the manufacturer’s instructions, as a measure of ex vivo functionality. Briefly, effluent samples (*n* = 7 malignant, n = 7 benign), controls and standards (25 μl) were loaded in duplicate into a pre-coated 96 well plate, followed by 100 μl working concentration of conjugate reagent. The plate was incubated for 60 min at room temperature. Each well was washed 5 times with distilled water before TMB (3,3′,5,5′-Tetramethylbenzidine; 100 μl) reagent was added before a further 20-min incubation. The reaction was stopped by the addition of 100 μl stop solution (2 N Sulphuric acid [H_2_SO_4_] VWR, Leicestershire, UK) to each well. Absorbance was measured at 450 nm using a Multiskan plate reader (Thermo Scientific). T4 concentration in each sample was determined from the standard curve and normalised per mg of thyroid tissue.

## Results

**Fig. 2:**
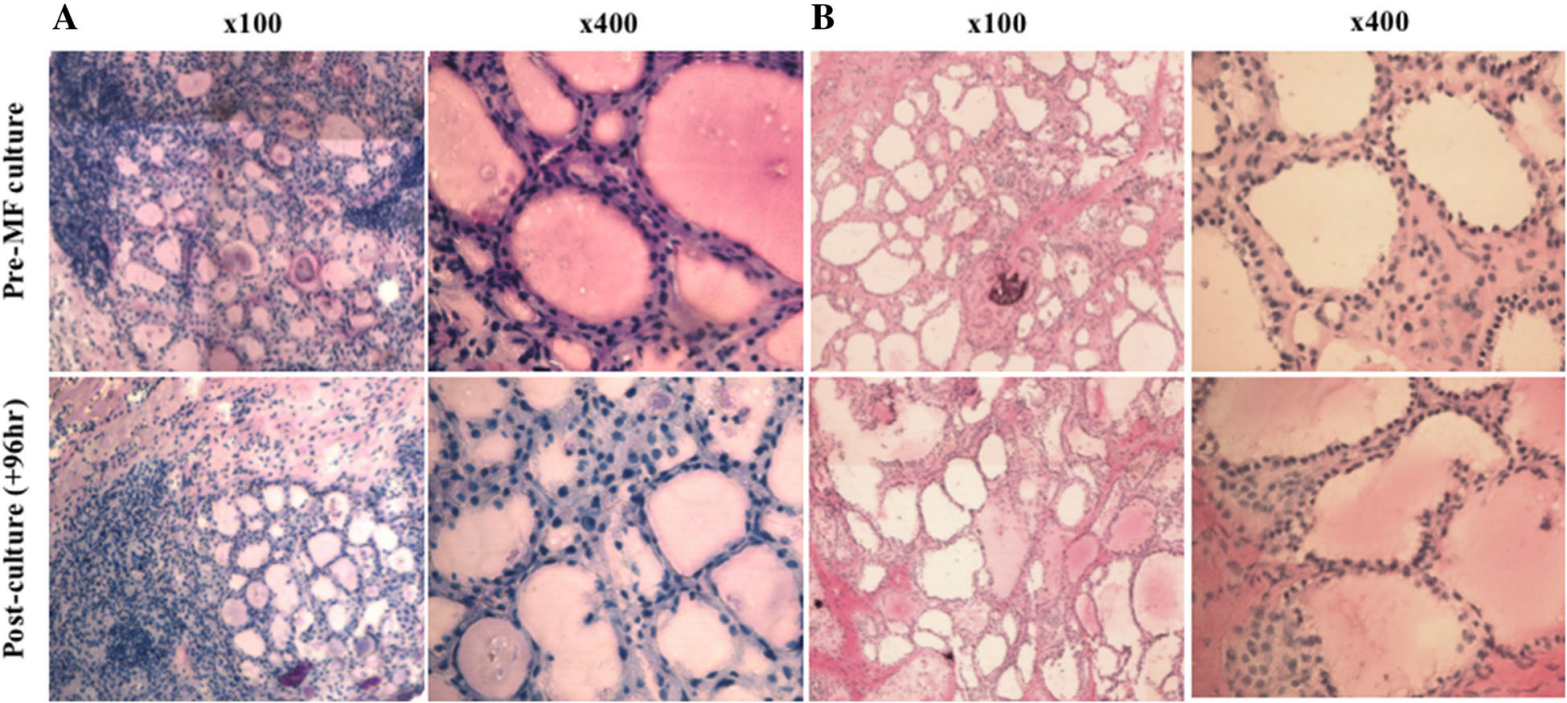
Haematoxylin and eosin stained 8 μM frozen thyroid sections. Representative images from two patients **a** and **b**. Top: fresh tissue frozen prior to culture. Bottom: tissue frozen subsequent to 96 h culture within the PCTS device. Images are shown at both 100x and 400x magnification

The morphology of both freshly resected and post-culture (96 h) ex vivo thyroid tissue sections was assessed by H&E staining and showed preservation of gross morphology, with retention of tissue cohesion (Fig. 2). The follicular structure, typical of in vivo thyroid tissue, was clearly displayed in both fresh and post-culture tissue samples. Inter-tumour variation in cellularity and follicle size is clear, however these differences are retained in tumour tissue throughout culture.

**Fig. 3:**
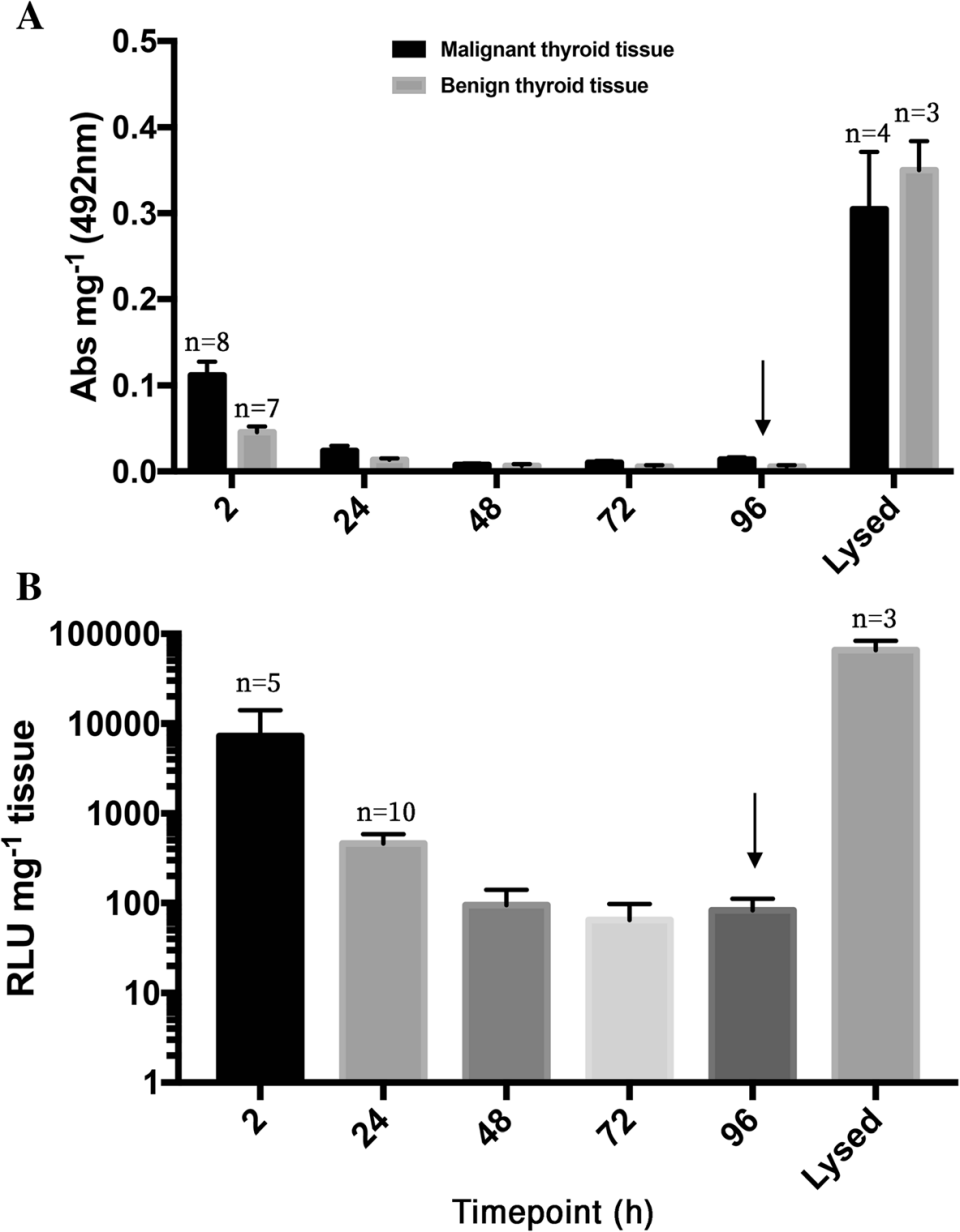
Measurement of lactate dehydrogenase (LDH) and dead-cell protease (DCP) release into culture effluent for the evaluation of cell membrane damage. **a**: Lactate dehydrogenase release from both malignant and benign thyroid tissue (*n* = 14 and *n* = 10, respectively unless otherwise stated; relative absorbance of formazan reaction product) during on-chip culture of thyroid tissue normalised per mg starting tissue wet weight. **b**: Level of relative light units (RLU) produced as a result of cleavage of the luminogenic assay substrate by dead cell protease released per mg of on-chip thyroid tissue over the 96 h culture period (*n* = 11). Lysis buffer (10% *v*/v) was added to homogenised tissue after 96 h culture (arrow) in order to purposefully rupture cell membranes and induce complete release of remaining LDH/ DCP

Culture effluent was sampled throughout the 96 h culture period and examined for the presence of two markers of cell death, LDH and DCP. An initial raised level of LDH release was observed within the first 2 h of culture setup (Fig. 3a), which reduced after 24 h and remained consistently low for the remaining 72 h, until tissue was exposed to 10% (*v*/v) lysis buffer, which led to an average increase in LDH release from 0.01 ± 0.002 to 0.304 ± 0.06 and 0.005 ± 0.001 to 0.35 ± 0.03 absorbance units mg^− 1^ in malignant and benign thyroid tissue, respectively. No difference in LDH release was found between the malignant and the benign tissue. DCP release by cells in the culture system followed a profile similar to that of LDH release (Fig. 3b), with the level being relatively high in the first 2 h of culture before decreasing after 24 h. DCP release reached a minimum after 72 h, with only 41.53 ± 28.33 RLU mg^− 1^ detectable. Concordant with LDH release, purposeful lysis of cultured thyroid tissue subsequent to 96 h culture induced a > 1000-fold increase in detectable DCP.

**Fig. 4:**
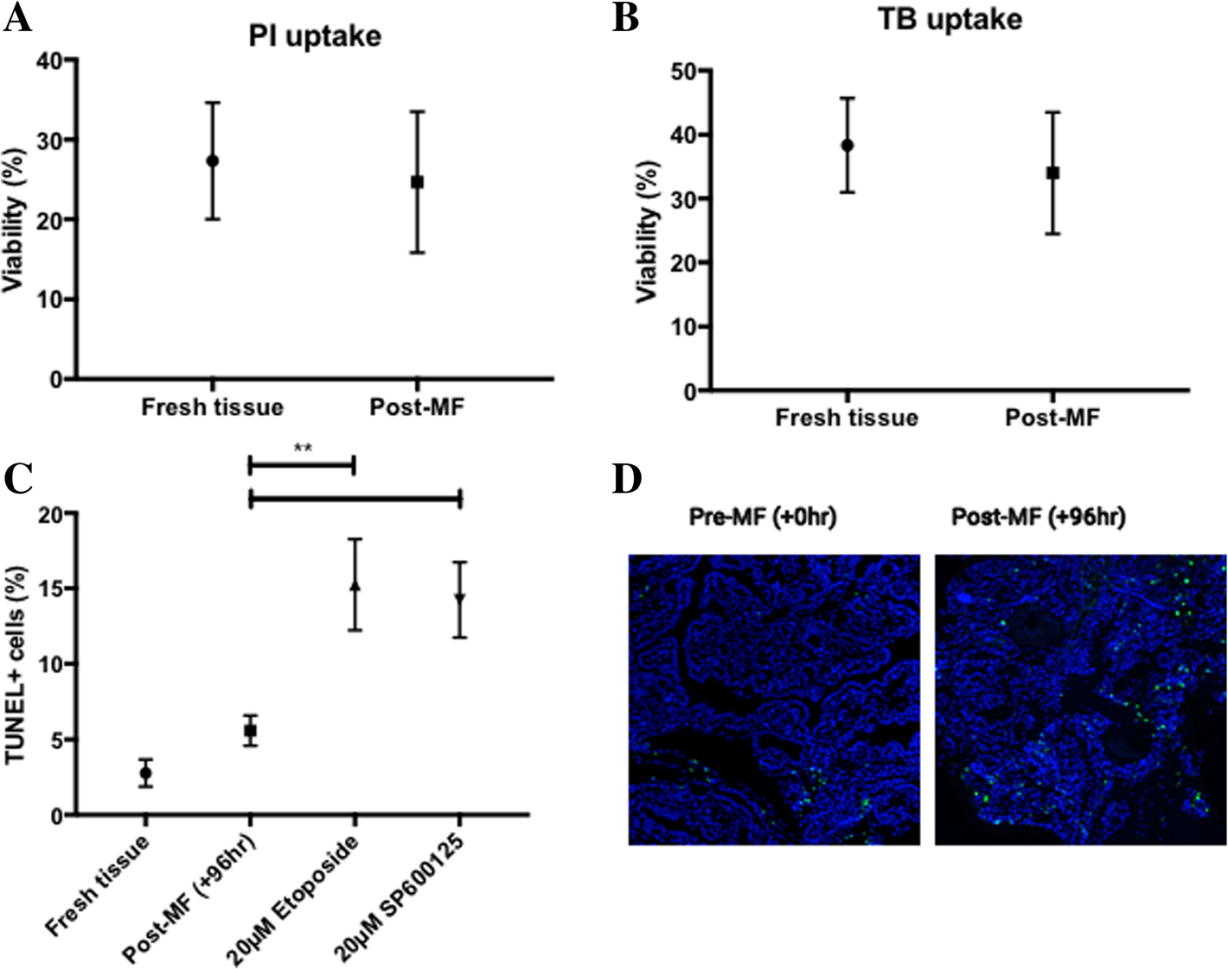
Cellular viability and apoptosis in freshly resected, control and treated thyroid tumour tissue. Cellular ability to prevent both PI (**a**) and TB (**b**) transit across the plasma membrane was employed as a proxy of viability (*n* = 3, mean + SEM). No significant difference was observed between fresh and untreated control thyroid tissue. **c**: Terminal deoxynucleotidyl transferase dUTP nick end labelling in both fresh (+ 0 h) and post-culture (+ 96 h) malignant thyroid tissue explants demonstrated no difference in the proportion of apoptotic cells in post-culture specimens (*n* = 9; 2.78 vs. 5.59%, *p =* 0.51 [paired t-test]*).* Treatment of cultured thyroid explants with etoposide and SP600125 resulted in a significant increase in apoptosis (15.25 and 14.24%, respectively) which were both significantly increased compared to untreated explants (*n* = 5; *p = <* 0.001). **d**: Representative images of both pre-MF and untreated post-MF thyroid explants illustrating apoptotic cells (FITC; green) counterstained with DAPI (blue)

No significant difference in viability between fresh (27.3 ± 7.3%) and post-96 h cultured (24.6 ± 8.8%) malignant thyroid tissue samples was observed using PI staining to (Fig. 4; *n* = 3; *p* = 0.19). These data were consistent with results using trypan blue exclusion to represent cell viability, whereby the mean proportion of viable dissociated cells was 38.33 ± 7.3% in fresh thyroid tissue prior to culture and 34 ± 9.5% in tissue after a 96 h culture period (Fig. 4; n = 3; *p* = 0.37). The proportion of apoptotic cells in both fresh and post-culture (+ 96 h) malignant thyroid tissues was assessed by TUNEL staining (*n* = 9) and a small, non-significant, increase in apoptotic cells was observed in the post-culture tissue (5.59 ± 0.99%) compared with the fresh tissue (2.77 ± 0.89%; Fig. 4-c and d). Moreover, on-chip treatment of thyroid explants with etoposide (topoisomerase II inhibitor) and SP600125 (JNK inhibitor) caused a significant increase in cellular apoptosis after 96 h treatment, compared to untreated thyroid tissue (15.25 and 14.24% vs. 5.59%; *p =* 0.0025 and *p =* 0.0069 respectively; Fig. 4).

**Fig. 5:**
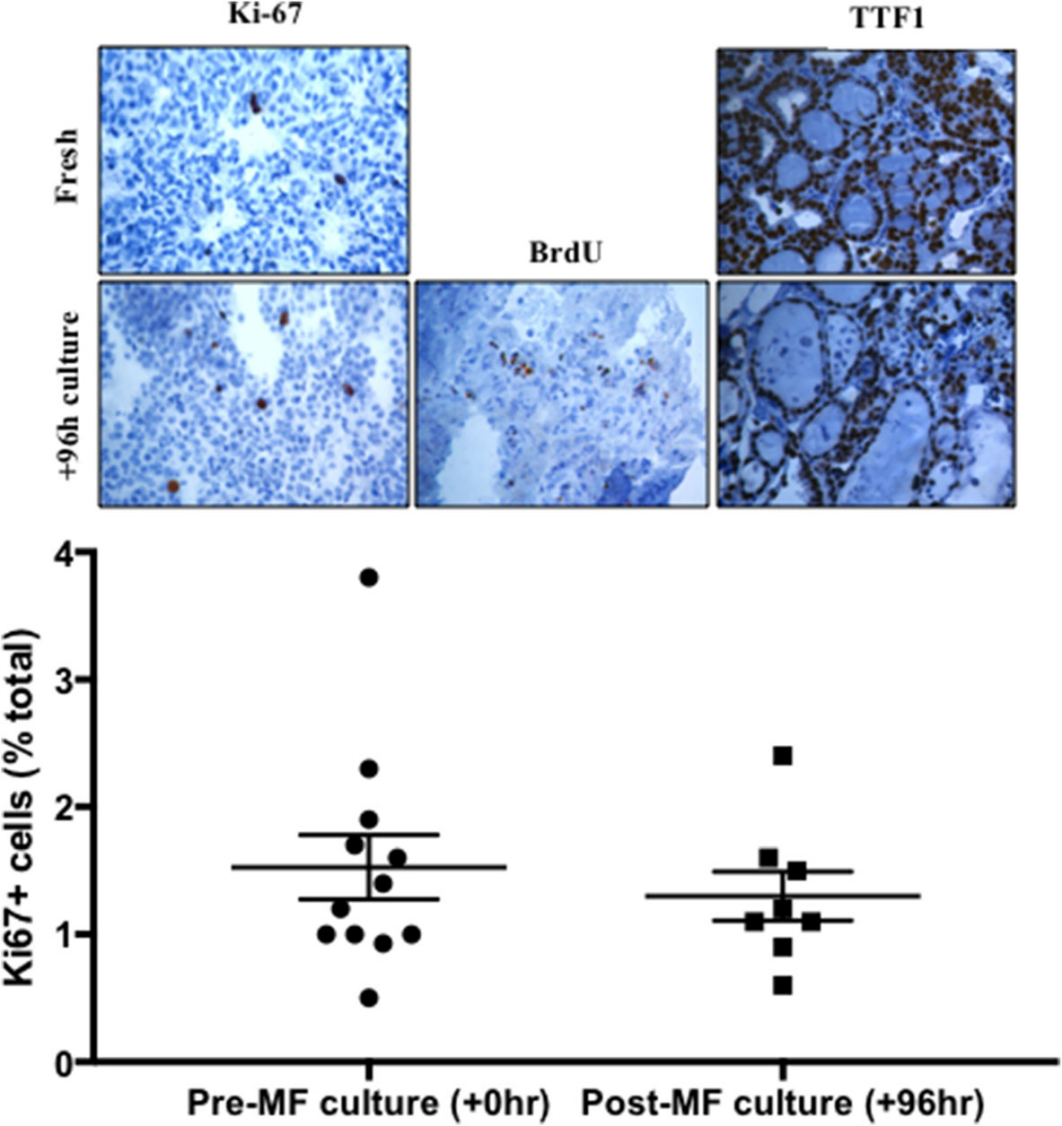
Percentage Ki-67 positivity in fresh (+ 0 h) and post-culture (+ 96 h) thyroid tumour tissue. Mean Ki-67 positivity was 1.52 ± 0.52% and 1.3 ± 0.34% in fresh and post-culture tissue, respectively; no significant difference was observed (*n* = 8; *p* = 0.32 [paired t-test]). Inset: Representative photomicrographs of Ki67, BrdU and TTF1 positive brown nuclei and counterstained with haematoxylin (× 400). BrdU staining is only shown in cultured tissue due to the requirement of perfusion with the molecule in order to elicit its incorporation and subsequent detection; 12.09 ± 4.38% cells stained positive for BrdU incorporation (n = 3)

Cell proliferation within the thyroid tumour tissue was investigated by IHC. Two markers were used; Ki67, a nuclear protein expressed during all active phases of the cell cycle (non-senescent cells) and BrdU which is incorporated during s-phase of the cell cycle [24]. This analysis demonstrated that proliferating cells were detectable in both freshly resected tissue and post-culture tissue. The mean percentage of Ki-67 positive nuclei was 1.45 ± 0.33% and 1.32 ± 0.34% in fresh and post-culture tissue (*n* = 8), respectively; no significant difference in the quantity of proliferating cells between fresh and post-culture malignant thyroid tissue was observed (*p* = 0.32). BrdU, which was perfused through the culture system for the final 24 h culture on the device, was clearly visible as incorporated into the cell nuclei of proliferating cells post-culture, illustrating that on-chip cell proliferation was occurring (Fig. 5). Subsequent to a 24 h perfusion with 10 μM BrdU, 12.09 ± 4.38% cells stained positive for BrdU incorporation (*n* = 3). Thyroid transcription factor 1 (TTF1) is crucial for thyroidal organogenesis and differentiation which, along with two other factors, paired box gene 8 (PAX8) and TTF2 (FOXE1), governs the expression of thyroglobulin (TG), thyroperoxidase (TPO) and NIS [25]. The expression of TTF1 was characterised in fresh (0 h) and post-culture (+ 96 h) malignant thyroid tissue; the staining pattern of TTF1 was maintained throughout culture in the microfluidic device (Fig. 5).

**Fig. 6:**
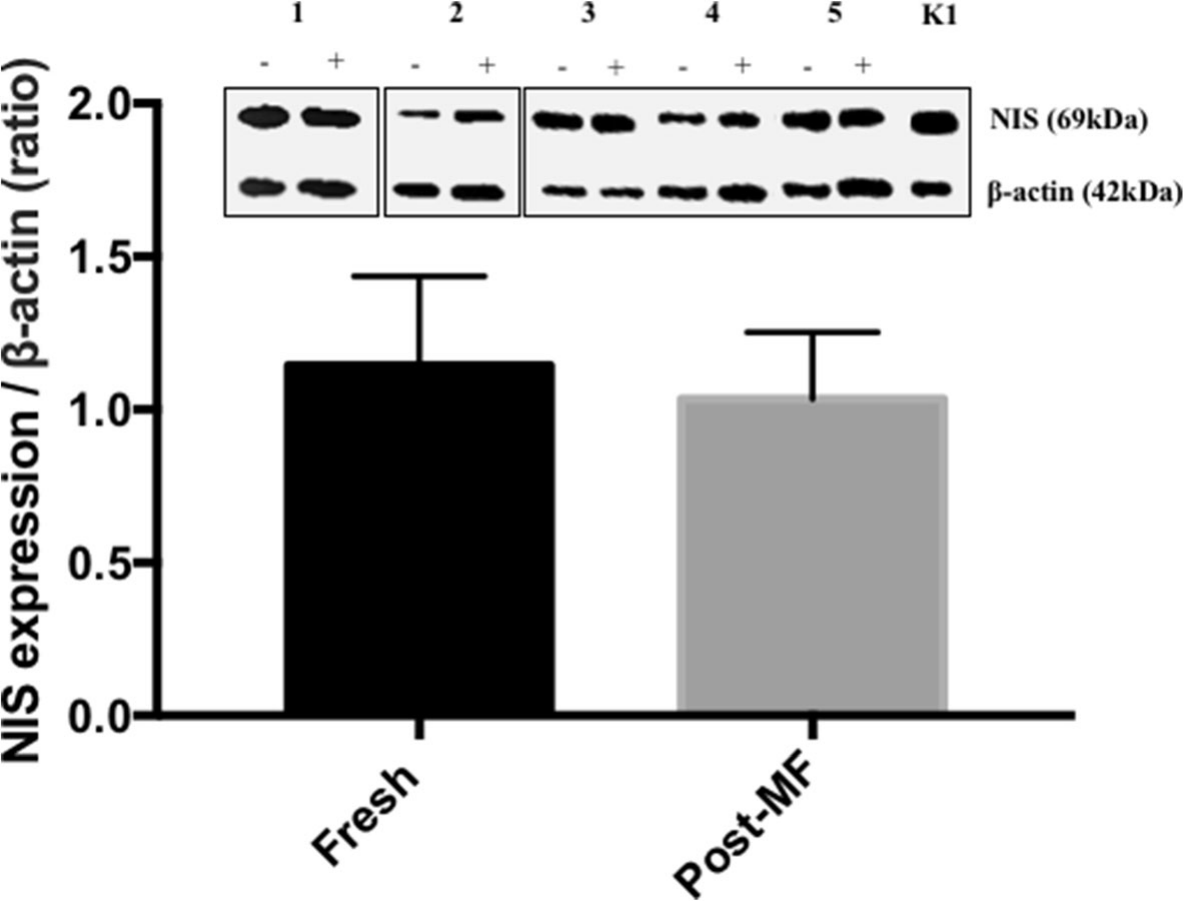
Assessment of sodium iodide symporter (NIS) protein expression by Western blotting. Band densitometry confirmed that NIS expression was not significantly altered by the microfluidic culture of thyroid tumour tissue explants (n = 5). [+] and [−] represent protein extracted from tumour tissue with and without on-chip culture respectively. 1–5 indicates five separate patient samples and K1 is protein extracted from the thyroid cell line

As expression of the NIS is crucial for adequate response to treatment to I^131^, it was important to ensure that 96 h culture of malignant thyroid explants within our MF device did not affect NIS protein levels. NIS expression was investigated in both freshly resected and post-culture malignant thyroid tissue and showed that there was no statistically significant difference between the density of NIS protein bands in fresh and cultured thyroid tissue, normalised to β-actin (Fig. 6; *n* = 5), indicating that the expression of NIS was maintained throughout culture.

**Fig. 7:**
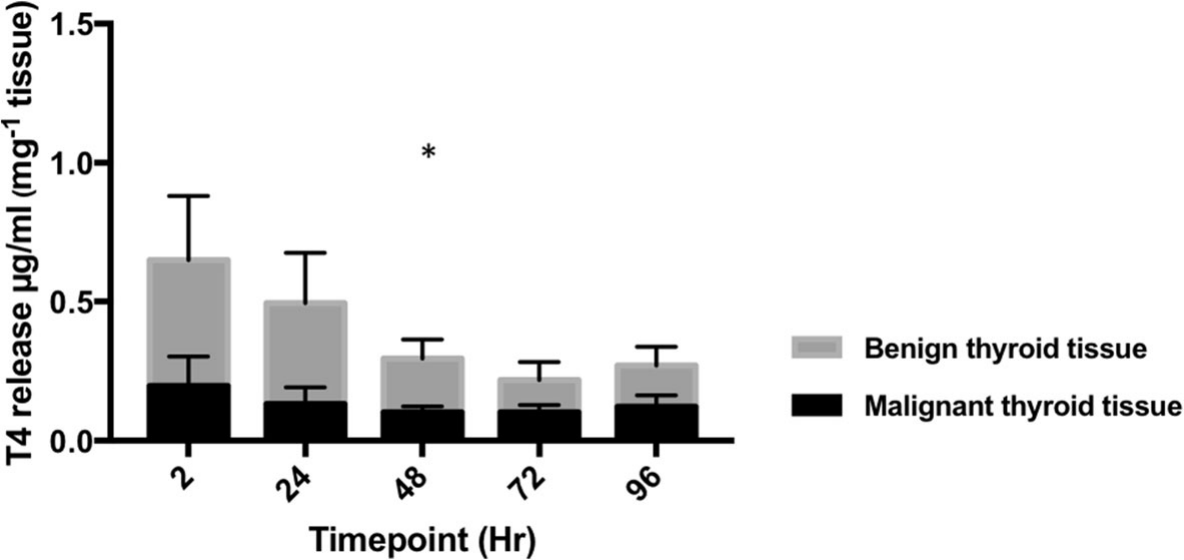
Thyroxine (T4) release by on-chip thyroid tissue. Concentration of T4 in effluent samples collected throughout the culture of both malignant (*n* = 7) and benign (n = 7) thyroid tissue explants on the PCTS device. Overall T4 release by benign thyroid tissue was significantly higher than that of malignant thyroid tissue (*p =* 0.03; 2-way ANOVA). Results normalised mg^− 1^ tissue

The functionality of cultured ex vivo thyroid tissue was investigated by its capability to synthesise and release the primary thyroid hormone, T4. Production of T4 occurs as a result of a cycle of biochemical reactions occurring exclusively within the thyroid. Culture effluent was analysed by ELISA for the presence of T4 (*n* = 7 malignant tissue; n = 7 benign tissue). Data indicate that T4 release by both malignant and benign thyroid explants follows a similar overall trend, with an initially raised level of T4 production over the first 24 h, stabilising after 48 h (Fig. 7). Interestingly, T4 release by benign thyroid explants was consistently higher at each timepoint in comparison to malignant tissue and average T4 output throughout the complete culture period was significantly higher in benign tissue (0.29 ± 0.09 μg/dl mg^− 1^) than in malignant tissue (0.14 ± 0.03 μg/dl mg^− 1^; Fig. 7; *p* = < 0.05).

## Discussion

The current study aimed to characterise the ability of a PCTS device to maintain viable thyroid tissue explants over a 96 h period with a view towards therapeutic interrogation. Human tissue explants from human head and neck tumours, intestine and mouse brain have been maintained using similar microfluidic culture systems [20, 26, 27] however this is, to the best of the authors’ knowledge, the first time ex vivo human thyroid tissue has been cultured in a robust, easy-to-use, platform. Tissue sections taken from both fresh and PCTS-cultured thyroid tissue analysed by haematoxylin and eosin and IHC staining demonstrated that morphology was preserved in tissue which had been cultured in the device for 96 h, validated by a consultant histopathologist. Results observed agree with those shown in similar studies from the group, whereby head and neck squamous cell carcinoma and full thickness intestinal tissue also demonstrated a preservation of tissue morphology after 72 h on-chip culture [18, 20]. The gross inter-patient morphology of tissue specimens was variable, and heterogeneity was visible within each tissue sample. Areas of typical thyroidal histology exhibiting follicular architecture often neighboured regions of densely arranged cells or fibrous stroma. This inter- and intra- patient heterogeneity was expected and was also presented in a study by Radu et al. [28], who speculated that the morphological variation in thyroid carcinoma is due to manifestation of diverse tumour cell clones under the influence of the same etio-pathogenic factors. It will therefore be necessary to run multiple PCTS chips from the same patient when interrogating the tissue with different treatment regimens.

The release of both LDH and DCP as markers of cell membrane integrity was assayed throughout the culture period; loss of cellular plasma membrane integrity is synonymous with loss of cellular viability [29] and microfluidic culture offers a unique means of evaluating this in real time. As anticipated, the release of these two markers followed similar patterns, with a higher level at the beginning of the culture period, attributed to the damage caused to the tissue during preparation, which decreased during the remainder of the experiment. The relatively low LDH/ DCP release once the tissue had recovered from the initial cellular damage during set-up, which has also been experienced in previous studies [30, 31], indicates cultured explants do not undergo further degradation due to factors such as sheer stress or lack of oxygen within the culture chamber. Deliberate lysis of cultured tissue resulted in an increase in both LDH and DCP, suggesting that a significant number of viable cells remain after 96 h in the PCTS device, which agrees with the morphological assessment. A similar pattern has been observed studies from other groups where stabilisation of LDH release was seen after four days in a four-organ-chip model [32] and after six days in a liver/skin co-culture [33]. The discrepancy in time taken for tissue to recover from the initial damage could potentially be attributable to either the difference in tissue type, the system used or the presence of malignancy in the current system.

Tissue dissociation using a combined mechanical and enzymatic method offered an alternative means of investigating the survival of individual cells constituting the gross thyroid explants, by monitoring the ability of propidium iodide and trypan blue to traverse the cellular membrane. Again, no statistically significant difference in mean cellular viability was observed between samples which had been dispersed prior to, or after PCTS-culture, further demonstrating that on-chip maintenance was not causing increased cell death. This was despite the fact that the overall viability of cells after dispersal from gross tumour material was unexpectedly low (< 40%), presumably due to injury caused by the enzymatic (collagenase IV, DNase II) and mechanical (mincing of gross tissue using scalpel blades) process of dissociation. The high level of cell death observed in the current study is in contrast to Li et al. [34] who provided a mean cell viability of 65.66% (±4.96) using trypan blue staining following disaggregation of murine pancreatic fragments using collagenase IV. As malignant tumour tissue is marked by regions of necrosis owing to rapid oncogene-driven proliferation not supported by an adequate vascular bed [35], it is a possibility that presence of necrotic cells contributed to the lower overall viability in the current study. The viability of cells dispersed from human head and neck cancer specimens was assessed by flow cytometry in a separate study and produced viabilities ranging from 24 to 92% [36], partially aligning with the results seen here.

To further characterise on-chip tissue kinetics, the proportion of thyroid cells undergoing apoptosis was elucidated through the labelling of 3-OH termini of DNA strand breaks in situ, produced due to endonuclease mediated DNA fragmentation, characteristic of DNA degradation and late-stage apoptosis [37]. The culture of thyroid explants within the microfluidic device showed no change in the proportion of apoptotic cells. Similar studies have, however, reported comparable levels of apoptosis in untreated specimens’ post-microfluidic culture. Cheah et al. [22], found an apoptotic fraction of < 10% within untreated head and neck squamous cell carcinoma (HNSCC), whilst Wang et al. [38] also saw < 10% apoptosis post-culture in brain organoids, compared to ~ 40% apoptosis in organoids subjected to static culture conditions. The increase in apoptosis observed in this study was not mirrored in other tests of viability such as PI/TB uptake and LDH/DCP leakage data as the latter assays rely on loss of membrane integrity, synonymous with necrotic cell death [38], to facilitate detection of leaked cellular components (LDH/DCP) or PI/TB labelled cells. It is feasible that, if cultured for an extended period of time, a decrease in cell viability would also be observed using PI/TB uptake and LDH/DCP leakage assays, due to the occurrence of cell death by alternative mechanisms such as secondary necrosis, characterised by progressive loss of plasma membrane integrity, through caspase-3-dependent cleavage of the GSDMD-like protein DFNA5 [38, 39]. Furthermore, on-chip treatment of thyroid explants with 20 μM etoposide and SP600125 demonstrates the devices’ utility as a drug screening system. Perfusion of thyroid explants with either agent for 96 h resulted in significantly increased levels of apoptosis as examined by TUNEL staining. Etoposide is a well-characterised inducer of apoptosis [40] whilst SP600125 is a multikinase inhibitor which has been shown to have both anti-proliferative and migratory effects in thyroid cell lines [41].

IHC analysis was used to investigate the ability of the cells within the cultured tissue to proliferate. The Ki67 antigen is present in all active stages of the cell cycle, except for G_0,_ in which cells are in a quiescent state [42]. Previous studies have attempted to use Ki67 labelling index to aid diagnosis in thyroid neoplasms, however it has shown little value in this setting due to the low labelling index in most thyroid lesions (< 1% in 57% PTC samples tested; [43]). In agreement, Ki67-positive brown stained cells were detectable at low levels (< 2%) in tissue sections in the current study after 96 h within the PCTS device indicating the presence of a small number of proliferating cells. Furthermore, comparison of the proliferating cell population in both fresh and post-culture thyroid tissue revealed no significant difference.

BrdU staining was also employed, having the added advantage of demonstrating definitive on-chip division as BrdU is incorporated into de novo DNA during the S-phase of the cell cycle as a substitute for thymidine and is detectable by IHC [44]. Cells with BrdU positive nuclei (12.09%) were evident in thyroid tissue samples which had been maintained for 96 h in the PCTS-culture device, the final 24 h of which included perfusion with 10 μM BrdU. Identification of BrdU-positive cells throughout the tissue both demonstrates the ability of BrdU to perfuse successfully through the tissue and moreover the presence of proliferating cells in the S-phase of the cell cycle. A comparable study by Sart et al. [45] investigated BrdU uptake in H4-II-EC3 rat hepatoma cells cultivated in both a 2D and 3D spheroid setting. The authors saw a near 2-fold decrease in BrdU positivity in 3D (~ 16%) versus 2D (~ 30%), similar to results observed in this study.

In addition to the viability of the tissue, it was important to characterise the differentiation of thyroid explants, in order to examine whether culture within the microfluidic device could affect the expression of certain genes crucial for successful treatment with radioiodine. A previous study conducted by Cirello et al. [46] which aimed to investigate the effects of the multikinase inhibitor SP600125 on multicellular spheroids derived from primary thyroid tissue, examined expression of the transcription factor TTF1 as a marker of differentiation. The authors observed maintenance of TTF1 expression in spheroids derived from primary thyroid tissue. Encouragingly, the pattern of TTF1 staining in post on-chip culture (+ 96 h) thyroid tissue was equivalent to that of fresh thyroid tissue, suggesting that culture for 96 h in the PCTS device did not markedly affect differentiation.

The NIS is an integral plasma membrane glycoprotein that facilitates the accumulation of I^−^ within the thyroid gland at a concentration up to 40 times higher than plasma [47]. Application of I^131^ for the targeted destruction of malignant thyroidal tissue has become one of the most effective radiation regimens available, causing fewer side effects than other cancer therapies [48]. Loss of NIS protein expression at the basolateral membrane of thyrocytes is a well-accepted precursor for reduced effectiveness of I^131^ therapy, as demonstrated in poorly differentiated and undifferentiated thyroid cancers [49, 50]. Therefore, it was necessary to assess the expression of NIS in thyroid explants which had been subjected to 96 h within the microfluidic device and elucidate whether its expression was altered. Western blot data revealed that there was no significant change in terms of NIS protein levels caused by culture within the PCTS device, indicating that the system is valid for the future testing of I^131^ sensitivity.

T4 is exclusively synthesised by thyrocytes as a pro-hormone, which becomes de-iodinated within target tissues to produce the more biologically potent T3 [51]. This natural phenomenon allowed the measure of T4 release from on-chip PCTS to be investigated as a measure of tissue functionality. Loss of, or reduced functional differentiation, occurs in all thyroid carcinomas leading to diminished expression of proteins central to thyroid hormone production [52]. In the current study, benign tissue produced a significantly higher volume of T4 than malignant tissue throughout the culture period, agreeing with the notion that malignant thyroid tissue has a decreased ability to produce hormone [11]. Detection of T4 within culture supernatant strongly suggests that thyroid hormone was synthesised de novo by on-chip thyroid tissue and that essential components such as I^−^ are reaching the thyrocytes. In the clinical setting, the thyroid hormone precursor TG is used as a specific marker of remnant tumour tissue after thyroid removal [53]. As our intention was to demonstrate thyroid tissue functionality, we decided to measure T4 levels as T4 synthesis relies on the success of additional mechanistic steps such as cellular I^−^ uptake and TG iodination.

## Conclusion

Results reported in this work validate the use of a PCTS device for the maintenance of ex vivo thyroid tissue which can be used for customising drug treatment. It is proposed to use the system described to delineate sensitivity of patients suffering from thyroid carcinoma to treatment with ^131^I, enabling patient stratification and reducing incidence of inappropriate treatment, reducing patient morbidity, whilst saving NHS resources. This is the first study of its kind to demonstrate reliably the maintenance of viable ex vivo thyroid tissue for up to 96 h, with pre- and post-culture tissue showing high uniformity in terms of morphology, viability and functionality.

## Abbreviations

BrdU: Bromodeoxyuridine
BSA: Bovine serum albumin
DAB: 3, 3 –diaminobenzidine
DCP: Dead cell protease; T4: thyroxine
DNA: Deoxyribonucleic acid
DTC: Differentiated thyroid carcinoma
ETFE: Ethylene tetrafluoroethylene
FTC: Follicular thyroid carcinoma
HCl: Hydrochloric acid
I^131^: Radioactive iodine
IHC: Immunohistochemistry
LDH: Lactate dehydrogenase
mRNA: Messenger ribonucleic acid
NIS: Sodium-iodide symporter
OCT: Optimal cutting temperature
PBS: Phosphate buffered saline
PCTS: Precision cut tissue slice
PEEK: Polyether ether ketone
PTC: Papillary thyroid carcinoma
RT-PCR: Real time-polymerase chain reaction
TC: Thyroid carcinoma
TF1: Thyroid transcription factor-1
TG: Thyroglobulin
TKI: Tyrosine kinase inhibitor
TMB: 3,3′,5,5′-Tetramethylbenzidine
TPO: Thyroid peroxidase

## References

[1] Kitahara CM, Sosa JA (2016). The changing incidence of thyroid cancer. Nat Rev Endocrinol.

[2] Fagin JA, Wells SA. Biologic and clinical perspectives on thyroid Cancer. NEJM. 2018;375(11):1054–67. 10.1056/NEJMra1501993 PMC5512163 27626519

[3] Darrouzet E, Lindenthal S, Marcellin D, Pellequer JL, Pourcher T (2014). The sodium/iodide symporter: state of the art of its molecular characterization. Biochim Biophys Acta.

[4] Kogai T, Brent GA (2012). The sodium iodide symporter (NIS): regulation and approaches to targeting for cancer therapeutics. Pharmacol Ther.

[5] Schlumberger M, Lacroix L, Russo D, Filetti S, Bidart JM (2007). Defects in iodide metabolism in thyroid cancer and implications for the follow-up and treatment of patients. Nat Clin Pract Endocrinol Metab.

[6] Min JJ, Chung JK, Lee YJ, Jeong JM, Lee DS, Jang JJ (2001). Relationship between expression of the sodium/iodide symporter and 131I uptake in recurrent lesions of differentiated thyroid carcinoma. Eur J Nucl Med.

[7] Schmidt A, Iglesias L, Klain M, Pitoia F, Schlumberger MJ (2017). Radioactive iodine-refractory differentiated thyroid cancer: an uncommon but challenging situation. Arch Endocrinol Metab.

[8] Albero A, Lopez JE, Torres A, de la Cruz L, Martin T (2016). Effectiveness of chemotherapy in advanced differentiated thyroid cancer: a systematic review. Endocr Relat Cancer.

[9] Schlumberger M, Tahara M, Wirth LJ, Robinson B, Brose MS, Elisei R, et al. Lenvatinib versus Placebo in Radioiodine-Refractory Thyroid Cancer. 2015. 10.1056/NEJMoa1406470. 10.1056/NEJMoa1406470 25671254

[10] Fallahi P, Mazzi V, Vita R, Ferrari SM, Materazzi G, Galleri D (2015). New therapies for dedifferentiated papillary thyroid cancer. Int J Mol Sci.

[11] Haugen BR, Alexander EK, Bible KC, Doherty GM, Mandel SJ, Nikiforov YE (2016). 2015 American Thyroid Association management guidelines for adult patients with thyroid nodules and differentiated thyroid Cancer: the American Thyroid Association guidelines task force on thyroid nodules and differentiated thyroid Cancer. Thyroid..

[12] Lee SJ, Choi KC, Han JP, Park YE, Choi MG (2007). Relationship of sodium/iodide symporter expression with I131 whole body scan uptake between primary and metastatic lymph node papillary thyroid carcinomas. J Endocrinol Investig.

[13] Arturi F, Russo D, Schlumberger M, du Villard JA, Caillou B, Vigneri P (1998). Iodide symporter gene expression in human thyroid tumors. J Clin Endocrinol Metab.

[14] Saito T, Endo T, Kawaguchi A, Ikeda M, Katoh R, Kawaoi A (1998). Increased expression of the sodium/iodide symporter in papillary thyroid carcinomas. J Clin Invest.

[15] Dohan O, Baloch Z, Banrevi Z, Livolsi V, Carrasco N (2001). Rapid communication: predominant intracellular overexpression of the Na(+)/I(−) symporter (NIS) in a large sampling of thyroid cancer cases. J Clin Endocrinol Metab.

[16] Russo E, Guerra A, Marotta V, Faggiano A, Colao A, Del Vecchio S (2013). Radioiodide induces apoptosis in human thyroid tissue in culture. Endocrine..

[17] Nagy N, Camby I, Decaestecker C, Chaboteaux C, Gras T, Darro F (1999). The influence of L-triiodothyronine, L-thyroxine, estradiol-17beta, the luteinizing-hormone-releasing hormone, the epidermal growth factor and gastrin on cell proliferation in organ cultures of 35 benign and 13 malignant human thyroid tumors. J Cancer Res Clin Oncol.

[18] Dawson A, Greenman J, Bower R, Green V (2018). Microfluidics: the fur-free way towards personalised medicine in cancer therapy. Drug Target Rev.

[19] Muskhelishvili L, Latendresse JR, Kodell RL, Henderson EB (2003). Evaluation of cell proliferation in rat tissues with BrdU, PCNA, Ki-67(MIB-5) immunohistochemistry and in situ hybridization for histone mRNA. J Histochem Cytochem.

[20] Bower R, Green VL, Kuvshinova E, Kuvshinov D, Karsai L, Crank ST (2017). Maintenance of head and neck tumor on-chip: gateway to personalized treatment?. Future Sci OA.

[21] Chan FK, Moriwaki K, De Rosa MJ (2013). Detection of necrosis by release of lactate dehydrogenase activity. Methods Mol Biol.

[22] Schindelin J, Arganda-Carreras I, Frise E, Kaynig V, Longair M, Pietzsch T (2012). Fiji: an open-source platform for biological-image analysis. Nat Methods.

[23] Tuominen VJ, Ruotoistenmaki S, Viitanen A, Jumppanen M, Isola J (2010). ImmunoRatio: a publicly available web application for quantitative image analysis of estrogen receptor (ER), progesterone receptor (PR), and Ki-67. Breast Cancer Res.

[24] Matatall KA, Kadmon CS, King KY (2018). Detecting hematopoietic stem cell proliferation using BrdU incorporation. Methods Mol Biol.

[25] Liu H, Lin F (2015). Application of immunohistochemistry in thyroid pathology. Arch Pathol Lab Med.

[26] Chang TC, Mikheev AM, Huynh W, Monnat RJ, Rostomily RC, Folch A (2014). Parallel microfluidic chemosensitivity testing on individual slice cultures. Lab Chip.

[27] Dawson A, Dyer C, Macfie J, Davies J, Karsai L, Greenman J, et al. A microfluidic chip based model for the study of full thickness human intestinal tissue using dual flow. Biomicrofluidics. 2016;10(6):064101. 10.1063/1.4964813 PMC5097047 27822333

[28] Radu TG, Ciurea ME, Mogoanta SS, Busuioc CJ, Grosu F, Tenovici M (2016). Papillary thyroid cancer stroma - histological and immunohistochemical study. Romanian J Morphol Embryol.

[29] Charlagorla P, Liu J, Patel M, Rushbrook JI, Zhang M (2013). Loss of plasma membrane integrity, complement response and formation of reactive oxygen species during early myocardial ischemia / reperfusion. Mol Immunol.

[30] Carr SD, Green VL, Stafford ND, Greenman J (2014). Analysis of radiation-induced cell death in head and neck squamous cell carcinoma and rat liver maintained in microfluidic devices. Otolaryngol Head Neck Surg.

[31] van Midwoud PM, Groothuis GM, Merema MT, Verpoorte E (2010). Microfluidic biochip for the perifusion of precision-cut rat liver slices for metabolism and toxicology studies. Biotechnol Bioeng.

[32] Maschmeyer I, Lorenz AK, Schimek K, Hasenberg T, Ramme AP, Hubner J (2015). A four-organ-chip for interconnected long-term co-culture of human intestine, liver, skin and kidney equivalents. Lab Chip.

[33] Wagner I, Materne EM, Brincker S, Sussbier U, Fradrich C, Busek M (2013). A dynamic multi-organ-chip for long-term cultivation and substance testing proven by 3D human liver and skin tissue co-culture. Lab Chip.

[34] Li D, Peng S, Zhang Z, Feng R, Li L, Liang J (2013). Complete disassociation of adult pancreas into viable single cells through cold trypsin-EDTA digestion*. J Zhejiang Univ Sci B.

[35] Eales KL, Hollinshead KE, Tennant DA (2016). Hypoxia and metabolic adaptation of cancer cells. Oncogenesis..

[36] Bijman JT, Wagener DJ, van Rennes H, Wessels JM, van den Broek P (1985). Flow cytometric evaluation of cell dispersion from human head and neck tumors. Cytometry..

[37] Darzynkiewicz Z, Galkowski D, Zhao H (2008). Analysis of apoptosis by cytometry using TUNEL assay. Methods..

[38] Yaqing W, Wang L, Guo Y, Zhu Y, Qin J (2018). Engineering stem cell-derived 3D brain organoids in a perfusable organ-on-a-chip system. RSC Adv.

[39] Rogers C, Fernandes-Alnemri T, Mayes L, Alnemri D, Cingolani G, Alnemri ES (2017). Cleavage of DFNA5 by caspase-3 during apoptosis mediates progression to secondary necrotic/pyroptotic cell death. Nat Commun.

[40] Karpinich NO, Tafani M, Rothman RJ, Russo MA, Farber JL (2002). The course of etoposide-induced apoptosis from damage to DNA and p53 activation to mitochondrial release of cytochrome c. J Biol Chem.

[41] Grassi ES, Vezzoli V, Negri I, Labadi A, Fugazzola L, Vitale G (2015). SP600125 has a remarkable anticancer potential against undifferentiated thyroid cancer through selective action on ROCK and p53 pathways. Oncotarget..

[42] Cattoretti G, Becker MH, Key G, Duchrow M, Schluter C, Galle J (1992). Monoclonal antibodies against recombinant parts of the Ki-67 antigen (MIB 1 and MIB 3) detect proliferating cells in microwave-processed formalin-fixed paraffin sections. J Pathol.

[43] Ito Y, Miyauchi A, Kakudo K, Hirokawa M, Kobayashi K, Miya A (2010). Prognostic significance of ki-67 labeling index in papillary thyroid carcinoma. World J Surg.

[44] Rew DA, Wilson GD (2000). Cell production rates in human tissues and tumours and their significance. Part II: clinical data. Eur J Surg Oncol.

[45] Sart S, Tomasi RF-X, Amselem G, Baroud CN (2017). Multiscale cytometry and regulation of 3D cell cultures on a chip. Nat Commun.

[46] Cirello V, Vaira V, Grassi ES, Vezzoli V, Ricca D, Colombo C (2017). Multicellular spheroids from normal and neoplastic thyroid tissues as a suitable model to test the effects of multikinase inhibitors. Oncotarget.

[47] Zicker S, Schoenherr B (2012). Focus on nutrition: the role of iodine in nutrition and metabolism. Compend Contin Educ Vet.

[48] Fisher DA (1996). Physiological variations in thyroid hormones: physiological and pathophysiological considerations. Clin Chem.

[49] Caillou B, Troalen F, Baudin E, Talbot M, Filetti S, Schlumberger M (1998). Na+/I- symporter distribution in human thyroid tissues: an immunohistochemical study. J Clin Endocrinol Metab.

[50] Park HJ, Kim JY, Park KY, Gong G, Hong SJ, Ahn IM (2000). Expressions of human sodium iodide symporter mRNA in primary and metastatic papillary thyroid carcinomas. Thyroid..

[51] Gerard AC, Daumerie C, Mestdagh C, Gohy S, De Burbure C, Costagliola S (2003). Correlation between the loss of thyroglobulin iodination and the expression of thyroid-specific proteins involved in iodine metabolism in thyroid carcinomas. J Clin Endocrinol Metab.

[52] Robbins RJ, Schlumberger MJ (2005). The evolving role of (131)I for the treatment of differentiated thyroid carcinoma. J Nucl Med.

[53] Luo Y, Ishido Y, Hiroi N, Ishii N, Suzuki K. The Emerging Roles of Thyroglobulin. Adv Endocrinol. 2014;2014(Article ID 189194):7.

